# Supplementary material for: Analysis of B-cell receptor repertoire to evaluate the immunogenicity of SARS-CoV-2 RBD mRNA vaccine: MAFB-7256a (DS-5670d)
Source: Front Immunol. 2024 Oct 7;15:1468760. doi: 10.3389/fimmu.2024.1468760 (PMC11491357; doi:10.3389/fimmu.2024.1468760)
Supplement: Supplementary file 1 [file Presentation1.pptx]

## Slide 1
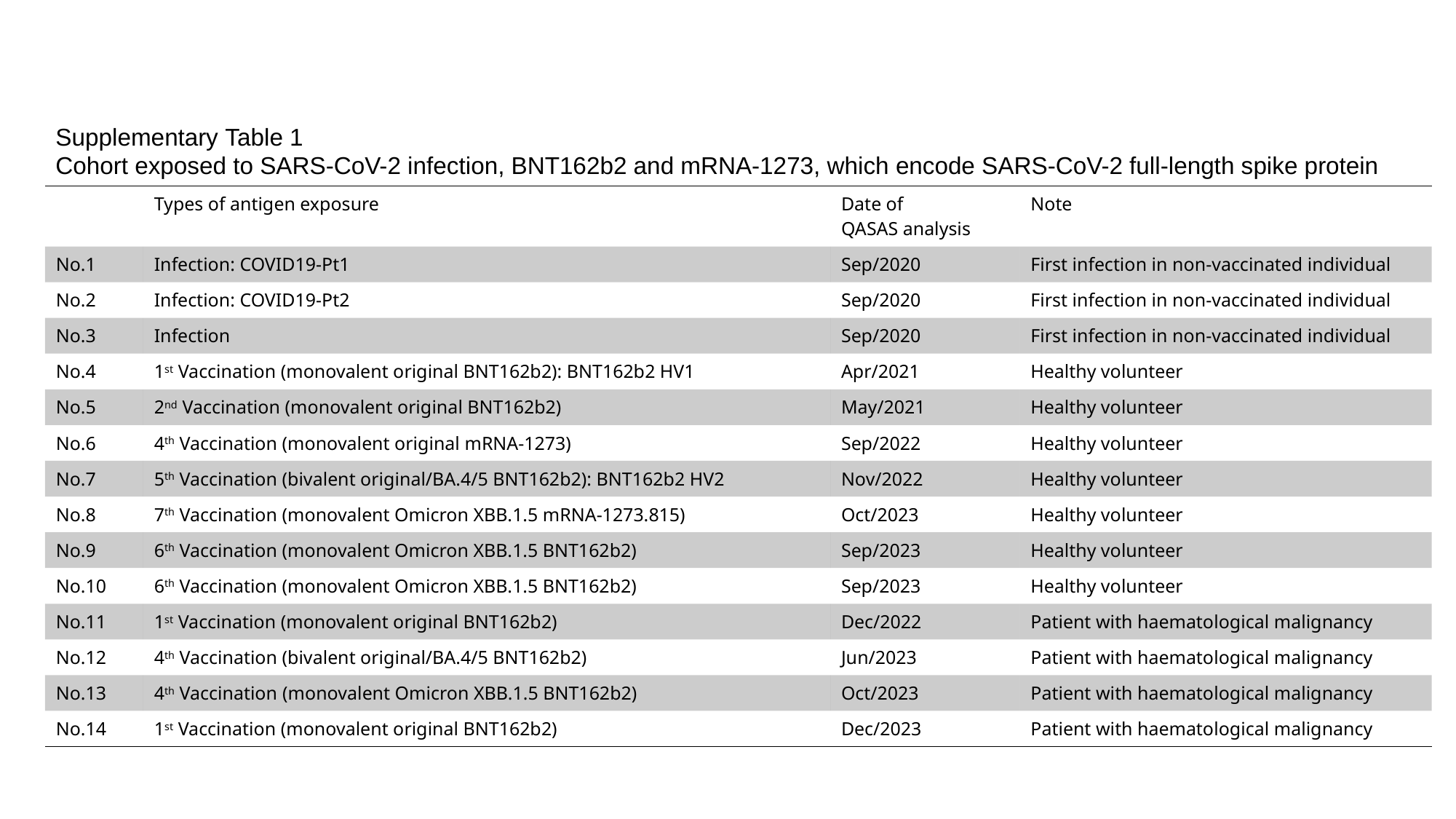

Supplementary Table 1
Cohort exposed to SARS-CoV-2 infection, BNT162b2 and mRNA-1273, which encode SARS-CoV-2 full-length spike protein
| | Types of antigen exposure | Date of QASAS analysis | Note |
| --- | --- | --- | --- |
| No.1 | Infection: COVID19-Pt1 | Sep/2020 | First infection in non-vaccinated individual |
| No.2 | Infection: COVID19-Pt2 | Sep/2020 | First infection in non-vaccinated individual |
| No.3 | Infection | Sep/2020 | First infection in non-vaccinated individual |
| No.4 | 1st Vaccination (monovalent original BNT162b2): BNT162b2 HV1 | Apr/2021 | Healthy volunteer |
| No.5 | 2nd Vaccination (monovalent original BNT162b2) | May/2021 | Healthy volunteer |
| No.6 | 4th Vaccination (monovalent original mRNA-1273) | Sep/2022 | Healthy volunteer |
| No.7 | 5th Vaccination (bivalent original/BA.4/5 BNT162b2): BNT162b2 HV2 | Nov/2022 | Healthy volunteer |
| No.8 | 7th Vaccination (monovalent Omicron XBB.1.5 mRNA-1273.815) | Oct/2023 | Healthy volunteer |
| No.9 | 6th Vaccination (monovalent Omicron XBB.1.5 BNT162b2) | Sep/2023 | Healthy volunteer |
| No.10 | 6th Vaccination (monovalent Omicron XBB.1.5 BNT162b2) | Sep/2023 | Healthy volunteer |
| No.11 | 1st Vaccination (monovalent original BNT162b2) | Dec/2022 | Patient with haematological malignancy |
| No.12 | 4th Vaccination (bivalent original/BA.4/5 BNT162b2) | Jun/2023 | Patient with haematological malignancy |
| No.13 | 4th Vaccination (monovalent Omicron XBB.1.5 BNT162b2) | Oct/2023 | Patient with haematological malignancy |
| No.14 | 1st Vaccination (monovalent original BNT162b2) | Dec/2023 | Patient with haematological malignancy |
