# Supplementary material for: Analysis of B-cell receptor repertoire to evaluate the immunogenicity of SARS-CoV-2 RBD mRNA vaccine: MAFB-7256a (DS-5670d)
Source: Front Immunol. 2024 Oct 7;15:1468760. doi: 10.3389/fimmu.2024.1468760 (PMC11491357; doi:10.3389/fimmu.2024.1468760)
Supplement: Supplementary file 2 [file Presentation2.pptx]

## Slide 1
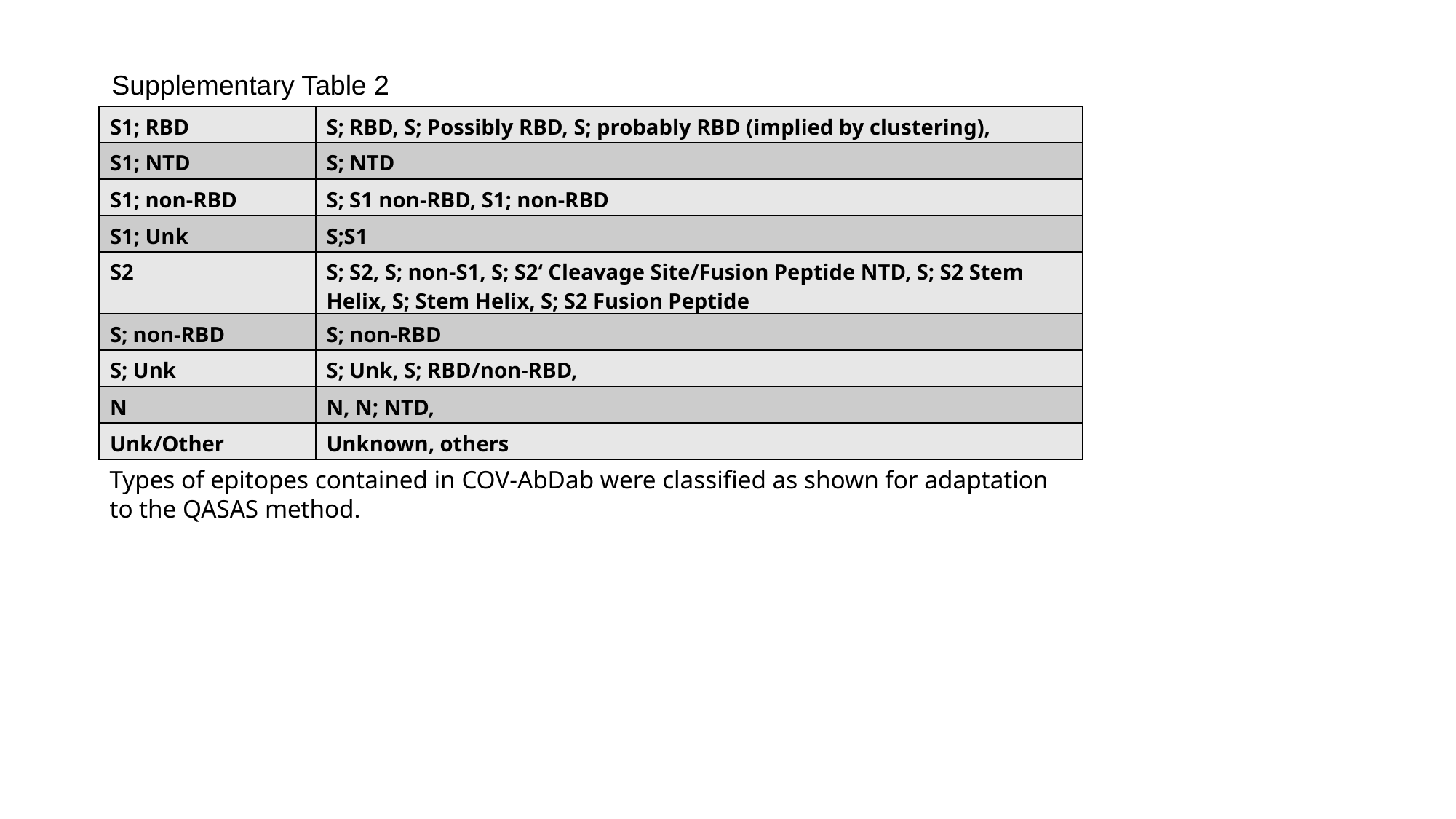

Supplementary Table 2
| S1; RBD | S; RBD, S; Possibly RBD, S; probably RBD (implied by clustering), |
| --- | --- |
| S1; NTD | S; NTD |
| S1; non-RBD | S; S1 non-RBD, S1; non-RBD |
| S1; Unk | S;S1 |
| S2 | S; S2, S; non-S1, S; S2‘ Cleavage Site/Fusion Peptide NTD, S; S2 Stem Helix, S; Stem Helix, S; S2 Fusion Peptide |
| S; non-RBD | S; non-RBD |
| S; Unk | S; Unk, S; RBD/non-RBD, |
| N | N, N; NTD, |
| Unk/Other | Unknown, others |
Types of epitopes contained in COV-AbDab were classified as shown for adaptation to the QASAS method.
