# Supplementary material for: Analysis of B-cell receptor repertoire to evaluate the immunogenicity of SARS-CoV-2 RBD mRNA vaccine: MAFB-7256a (DS-5670d)
Source: Front Immunol. 2024 Oct 7;15:1468760. doi: 10.3389/fimmu.2024.1468760 (PMC11491357; doi:10.3389/fimmu.2024.1468760)
Supplement: Supplementary file 3 [file Presentation3.pptx]

## Slide 1
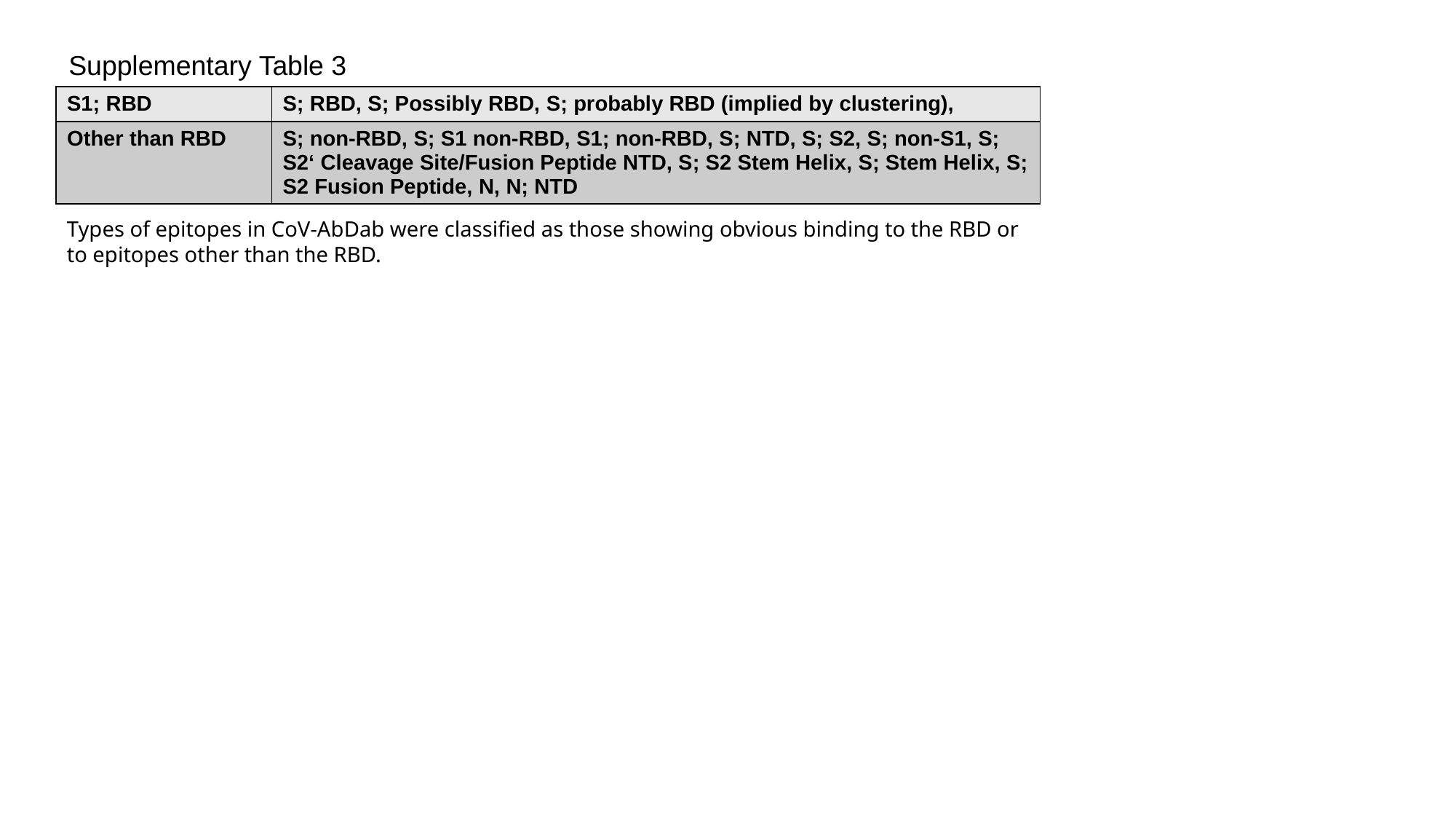

Supplementary Table 3
| S1; RBD | S; RBD, S; Possibly RBD, S; probably RBD (implied by clustering), |
| --- | --- |
| Other than RBD | S; non-RBD, S; S1 non-RBD, S1; non-RBD, S; NTD, S; S2, S; non-S1, S; S2‘ Cleavage Site/Fusion Peptide NTD, S; S2 Stem Helix, S; Stem Helix, S; S2 Fusion Peptide, N, N; NTD |
Types of epitopes in CoV-AbDab were classified as those showing obvious binding to the RBD or to epitopes other than the RBD.
